# MCPIP1 Down-Regulates IL-2 Expression through an ARE-Independent Pathway

**DOI:** 10.1371/journal.pone.0049841

**Published:** 2012-11-21

**Authors:** Min Li, Wenqiang Cao, Haifeng Liu, Wei Zhang, Xia Liu, Zhijian Cai, Jing Guo, Xuelian Wang, Zhaoyuan Hui, Hang Zhang, Jianli Wang, Lie Wang

**Affiliations:** 1 Institute of Immunology, Zhejiang University School of Medicine, Hangzhou, China; 2 Laboratory of Molecular Cell Biology, Institute of Biochemistry and Cell Biology, Chinese Academy of Sciences, Shanghai, China; German Cancer Research Center, Germany

## Abstract

IL-2 plays a key role in the survival and proliferation of immune cells, especially T lymphocytes. Its expression is precisely regulated at transcriptional and posttranscriptional level. IL-2 is known to be regulated by RNA binding proteins, such as tristetraprolin (TTP), via an AU-rich element (ARE) in the 3′-untranslated region (3′UTR) to influence the stability of mRNA. MCPIP1, identified as a novel RNase, can degrade IL-6, IL-12 and TNF-α mRNA by an ARE-independent pathway in the activation of macrophages. Here, we reported that MCPIP1 was induced in the activation of T lymphocytes and negatively regulated IL-2 gene expression in both mouse and human primary T lymphocytes through destabilizing its mRNA. A set of Luciferase reporter assay demonstrated that a non-ARE conserved element in IL-2 3′UTR, which formed a stem-loop structure, responded to MCPIP1 activity.RNA immunoprecipitation and Biotin pulldown experiments further suggested that MCPIP1 could modestly bind to IL-2 mRNA. Taken together, these data demonstrate that MCPIP1 down-regulates IL-2 via an ARE-independent pathway.

## Introduction

Interleukin 2 (IL-2), a key T lymphocytes-derived immuno-regulatory cytokine, plays a major role in maintaining lymphocyte homeostasis [1]. IL-2 stimulates the activation, survival and proliferation of T lymphocytes *in vitro* [1], while its main function *in vivo* is to limit lymphoid expansion and to promote peripheral tolerance [2], [3]. The absence of IL-2 results in the development of lethal autoimmunity and abnormal high level of IL-2 impairs the functions of many immune cells [4], [5].

IL-2 mRNA is rarely expressed in resting T lymphocytes, but is quickly induced after stimulation by integrated signals from T cell receptor (TCR) and CD28 co-receptor, or by phorbol ester (PMA) [6]. The IL-2 mRNA achieves its normal level in the stimulated T lymphocytes by regulating the rate of transcriptional activation and mRNA degradation. The transcription of IL-2 is regulated mainly through its promoter region, which is the binding site for transcription factors, such as activating protein-1 (AP-1) and NF-κB [6]. The posttranscriptional regulation is via controlling the stability of mRNA. The AU-rich element (ARE), which contains a core sequence of AUUUA in the 3′ untranslated region (UTR ), is the most common element for affecting mRNA stability via the binding of RNA binding proteins or microRNAs [7], [8]. Tristetraprolin (TTP, also known as Zfp36) is one of the most widely studied RNA binding proteins. It was identified as a regulator involved in the turnover of mRNAs of cytokines, such as, TNF-α [9], IL-2 and IL-17 [10], [11], in an ARE dependent manner. On the contrary, Hu antigen R (HuR ) and Nuclear factor 90 (NF90) are also RNA-binding proteins which bind to IL-2 3′UTR but increase its mRNA stability [12], [13].

The CCCH zinc finger protein MCPIP1 (encoded by *ZC3H12A* gene) is previously known as a negative regulator in the macrophage activation [14], [15], [16]. It has been recently identified in human peripheral blood monocytes induced by the monocyte chemotactic protein (MCP). Further studies demonstrate that the MCPIP1 gene can be significantly induced by proinflammatory molecules such as TNF-α, MCP-1 and IL-1β, implying that MCPIP1 may play a key role in inflammation [16]. *MCPIP1* knockout mice developed a syndrome of severe anemia or severe autoimmune response, such as augmented serum immunoglobulin levels, autoantibody production and increased plasma cells; and most of the mice died within 12 weeks [17], [18]. The underlying mechanism that *MCPIP1* negatively regulates inflammatory factors has been studied at different levels. One study shows that MCPIP1 acts as a deubiquitinase to negatively regulate JNK and NF-κB signaling by targeting the TNF receptor-associated factors (TRAFs) [18]. Other studies suggest that MCPIP1 is an essential RNase and down-regulates specific mRNAs of cytokines, including IL-6 and IL12p40, via a conserved region in the non-ARE of the 3′UTR [17]. Furthermore, MCPIP1 recognizes a stem-loop structure in the precursor-miRNA (pre-miRNA) and antagonize Dicer function to suppress miRNA biogenesis [19].

The RNA binding proteins TTP, HuR and NF90 have been reported to be expressed in T lymphocytes and regulate IL-2 mRNA in an ARE-dependent pathway. Sequence analysis shows the presence of the conserved non-ARE elements in the IL-2 mRNA 3′UTR, suggesting that the non-ARE elements may also be involved in the IL-2 posttranscriptional regulation. It has been reported that MCPIP1 negatively regulates IL-6 and IL-12 mRNA by recognizing a stem-loop structure in a non-ARE region, so we hypothesize that MCPIP1 may participate in the IL-2 posttranscriptional regulation in an ARE-independent pathway. Overexpression and knockdown analysis showed that MCPIP1 negatively regulated the IL-2 level in the primary CD4^+^T lymphocytes of both mice and human and this effect was partly due to affect the decay rate of IL-2 mRNA. Our Luciferase reporter assays demonstrated that a conserved non-ARE specific element in *lL-2 3′*UTR, which formed a stem-loop structure, responded to MCPIP1 activity. We also revealed that MCPIP1 modestly bound to IL-2 3′UTR. Taken together, our data suggest that MCPIP1 plays an important role in the down-regulation of IL-2 in the activated T lymphocytes by an ARE-independent pathway.

## Materials and Methods

### Ethics Statement

The animal experiments were performed in accordance with Zhejiang University institutional guidelines, and the study was approved by the Ethics Committee of Zhejiang University in written form. Euthanasia of mice was performed by carbon dioxide inhalation with minimum fear, anxiety and pain.

Human peripheral blood was obtained from healthy staff and students at the Zhejiang University School of Medicine. This study was approved by the Ethics Committee of Zhejiang University in written form and written informed consents were obtained from all volunteers prior to the experiments.

### Mice and Reagents

C57BL/6 mice (∼6-8 weeks old) were purchased from Shanghai Slac Animal Inc. (Shanghai, China) and housed in Experimental Animal Center of Zhejiang University. Anti-MCPIP1 polyclonal antibody (sc-136750), anti-β-actin and peroxidase (HRP)-labeled secondary antibody were purchased from Santa Cruz Biotec (Santa Cruz, CA). Anti-flag antibody (F1804) was purchased from Sigma (St. Louis, MO). Lipofectamine 2000 was purchased from Invitrogen (Grand Island, NY). Anti-human CD3 (16-0037-85), anti-human CD28 (16-0289-85), anti-mouse CD3e (16-0031-86) and anti-mouse CD28 (16-0281-86) antibodies were purchased from eBioscience (San Diego, CA). Human lymphocyte separation medium (LTS1077) was purchased from Jin Haoyang Biological manufacture (Tianjin, China). PMA (Cat.No.P-8139) and ionomycin (Cat.No.I-0634) were purchased from Sigma.

### Cell Culture

HEK293 (CRL-1573) and EL-4 (TIB-39) cell lines were purchased from American Type Culture Collection (ATCC) (Manassas, VA) and cultured in Dulbecco's Modified Eagle's Medium supplemented with 2 mM glutamine, 100 units/ml penicillin and 100 µg/ml streptomycin sulfate, and 10% heat-inactivated fetal bovine serum (FBS) (GIBCO) at 37°C in the presence of 5% CO_2_.

Mouse CD4^+^T lymphocytes were purified from the spleen and lymph nodes of 4∼8-week-old C57BL/6 mice (≥95% pure) using Dynal Mouse CD4^+^T negative Isolation Kit (Invitrogen Cat.no.114.15D). Human peripheral blood mononuclear cells (PBMC) were obtained from healthy subjects by Ficoll-Hypaque density centrifugation, as indicated previously [20]. The human blood CD4^+^T lymphocytes were isolated using Dynal Untouched Human CD4^+^T lymphocytes Kit (Invitrogen Cat.no.113.46D). These cells were cultured with anti-CD3 (1 µg/ml) and anti-CD28 (3 µg/ml) antibodies for the indicated time in 10% FBS-RPMI 1640 media with 2 mM glutamine, 10mM HEPES buffer, 100 units/ml penicillin and 100 µg/ml streptomycin.

### Plasmids

Recombinant vector encoding mouse *MCPIP1* (GenBank Accession number NM_153159) was constructed by PCR-based amplification and subcloned into the pCMV-Tag2b fused with a flag tag by using EcoRI and HindIII. Human *MCPIP1* (GenBank Accession number NM_025079) was cloned into pCMV-Tag2a using the same protocol above. For the transient transfection experiment, pCMV-Tag2a/b was used as a control. The primers were as followed:

mMCPIP1-flag:

GAATTCATGAGTGACCCTTGTGG, AAGCTTCCGACAGCCCCTTGC

hMCPIP1-flag:

GAATTCA ATG AGTGGCCCCT, AAGCTTATTACTCACTGGGGTGCTG

### Luciferase Reporter Assay

The 3′UTR luciferase reporter vectors containing full length of *IL-2*-3′UTR, *IL-6*-3′UTR, *IL-12p40*-3′UTR, *IFN-γ*-3′UTR; and parts of *IL-2*-3′UTR were generated by amplifying the corresponding fragments into XbalI site of PGL3-prooter vector (Promega, Madison, WI).

HEK-293 cells were cotransfected with 100 ng luciferase reporter plasmid, 10 ng thymidine kinase promoter-Renilla luciferase reporter plasmid, and the *mMCPIP-flag* or control vector. After 48 h, luciferase activities were determined by the Dual-Luciferase Reporter Assay System (Promega, Cat.No.E10910) according to the manufacturer’s instructions.

The primers were as followed:

IL-2-3′UTR:

CTATGTACCTCCTGCTTA, TTTTTTTTTAGAGGAGAGC

IL-6-3′UTR:

TAGTGCGTTATGCCTAAGCA, GTTTGAAGACAGTCTAAACAT

IL-12p40-3′UTR:

GATGCAACGTTGGAAAGG, TGTTTTTGAGACAGAATTTCTG

IFN-γ-3′UTR:

TTCGGGGTGGGGAAGAGATTG, GGTTGCAAAGGTATACTTTAT

IL-2-3′UTR (1-82):

CTATGTACCTCCTGC, AAACAATACATCCAAA

IL-2-3′UTR (83-166):

ACTATCTTTTGTAACTACTA, CATTTTTGAGCCCTTGG

IL-2-3′UTR (167-254):

TTTAAACTATTTATCTGAAA, TCATCTAAATACTTTTATTA

IL-2-3′UTR (255-391):

TCAAATATAAATAAGCTCA, TTTTTTTTTAGAGGAGAG

### Transfection

Transient transfection of mouse or human primary CD4^+^T lymphocytes was performed by electroporation following the manufacturer’s instruction (Amaxa Mouse T cell Nucleofector Kit, Cat.No.VPA-1006; Human T cell Nucleofector Kit, Cat. No.VPA-1002). Briefly, isolated CD4^+^ T lymphocytes were resuspended in electroporation buffer. After electroporation, cells were incubated for 4 h and then were stimulated with or without anti-CD3 (1 µg/ml) and anti-CD28 (3 µg/ml) for the indicated time.

Transient transfection into HEK293 cells was performed by Lipofectamine 2000 according to the manufacturer’s instruction.

### Short Interference RNA (siRNA) Knockdown

The pre-designed siRNA targeting to the mouse MCPIP1 (ID number 17084) and negative control were purchased from Invitrogen [16].

Human MCPIP1 siRNA targeting sequences were as follows:

Sense sequences:

5′-GUAAGAAGCCACUCACUUUUU-3′, 5′-GCAAGCGGGUGGUGUGCUAUU-3′,

5′-CCAACACGGUGCUGGGUGAUU-3′, 5′-AUACUAAGCUGUGUGGUGUUU-3′,

antisense sequences:

5′-AAAGUGAGUGGCUUCUUACUU-3′,

5′-UAGCACACCACCCGCUUGCUU-3′, 5′-UCACCCAGCACCGUGUUGGUU-3′,

5′-ACACCACACAGCUUAGUAUUU-3′

The above sequences were selected as reported previously [21] and were ordered from GenePharma Inc (Shanghai, China).

The siRNA was transfected into purified CD4^+^T lymphocytes by elecroporation using Amaxa Electroporation Unit following the manufacturer’s instruction.

### mRNA Decay Assay

To analyze the endogenous IL-2 mRNA decay rate, purified mouse T cells or human T cells were transient transfected with MCPIP1 siRNA and stimulated with anti-CD3 and CD28 Abs for 6 hours. Actinomycin D (10µg/ml) was added and total RNA was harvested after 0, 15, 30, or 45 min.IL-2 mRNA levels were measured by Q-PCR and normalized to β-actin. The normalized IL-2 mRNA level at time 0 was set at 100.

### RNA Isolation and Quantitative PCR (Q-PCR)

Total RNA was extracted using TRIzol reagent (Invitrogen, Cat.No. 15596026). Reverse transcription (Takara, Cat.No.DRR063A) and Q-PCR (Takara, Cat.No.DRR041A) were performed according to the manufacture’s instruction. Q-PCR was performed on a 7500 Real-Time PCR system (Applied Biosystems, Carlsbad, CA).

The corresponding primers were as follows:

mouseMCPIP1:

TCCGACGGAGTGGTGGTC, TGTGCTCAGAAGGCAGTGG;

humanMCPIP1:

AACTGGAGAAGAAGAAGATCCTGG,

ATTGACGAAGGAGTACATGAGCAG;

mouse-β-actin:

aacagtccgcctagaagcac, cgttgacatccgtaaagacc;

human- β-actin:

TGGAGAAAATCTGGCACCACACC,

GATGGGCACAGTGTGGGTGACCC;

mouse-IL-2:

GATGAACTTGGACCTCTGCG, CATCATCGAATTGGCACTCA;

human-IL-2:

ACTCAAACCTCTGGAGGAAGTGCT, ACAATGGTTGCTGTCTCATCAGCAT.

### Western Blot

Total cells were lysed in complete lysis-M buffer (Roche; Cat.No. 04719956001) and the concentration was determined by the bicinchoninic acid (BCA) protein assay (Themo; Lot # MC 155209). Equal amount of protein was subjected to SDS-PAGE, transferred onto nitrocellulose membrane, and hybrid-blotted as described previously [22].

### Measurement of Cytokine

IL-2 concentration in cell culture supernatants was measured using enzyme-linked immunosorbent assay kits (eBioscience; Mouse: Cat.No. 88-7024-22; Human: Cat.No. 88-7025-86).

### RNA Immunoprecipitation

EL-4 cells were transfected with mMCPIP1-flag or control plasmid. After 24 h, cells were stimulated with PMA (5 ng/ml) and ionomycin (500 ng/ml) for 3 h. Cell lysates were subjected to RIP with monoclonal antibody against flag or IgG according to the manufacture’s instruction (Millipore; Cat.No. 17-701). Purified mouse T cells were stimulated with anti-CD3 (1 µg/ml) and anti-CD28 (3 µg/ml) antibodies for 6 h. Cell lysates were subjected to RIP with anti-MCPIP1 antibody or IgG. Immumoprecipitated RNA was reverse transcribed into cDNA, then quantified using Sybr Green qPCR or semi-quantitative PCR. Data were expressed as fold enrichment of anti-flag Ab relative to IgG.

### In vitro Biotin Pulldown Assay

IL2-3′UTR, IL2 3′UTR (Δ83-166) or IL2-3′UTR mutant1 were generated by RT-PCR of pGL3-il2-3′UTR or pGL3-il2-3′UTR-mutant1 for transcripts using forward primers that contained a T7 transcription initiation site.

(5-CCAAGCTTCTAATACGACTCACTATAGGGAGA-3). PCR products were purified and used as templates for the synthesis of biotinylated RNA by biotin-conjugated UTP and T7 RNA polymerase according to the manufacturer’s instructions (Roche, Cat. No.10881767001). HEK293 cells were transfected with MCPIP1-flag plasmid and after 48 h cell lysates were prepared and incubated for 1 h at room temperature with 1 µg biotinylated transcripts, then RNA-protein complexes were incubated with streptavidin-conjugated Dynabeads (Invitrogen, Cat. No.653.05). Protein in the pulldown beads was detected by Western blot with anti-flag antibody as described [23].

### Statistic Analysis

Data were presented as the mean ± SD. Statistical significance was determined by Student’s *t*-test. P<0.05 was considered to be statistically significant.

## Results

### Induction of MCPIP Proteins during the Primary T lymphocytes Activation

**Figure 1:**
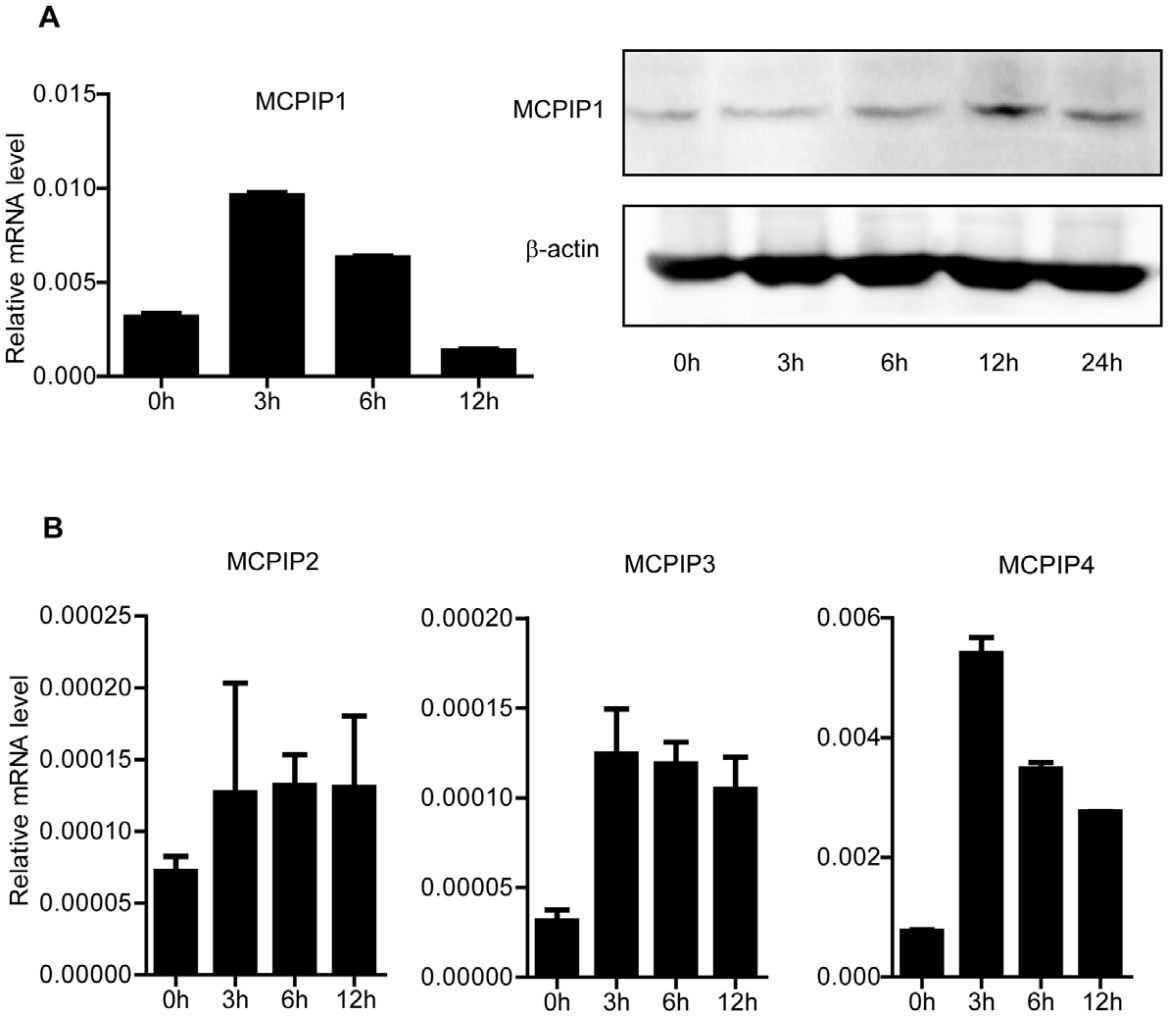
MCPIP1 proteins are induced by anti-CD3 and anti-CD28 Abs in mouse primary CD4^+^T lymphocytes. (A). Detection of MCPIP1 level by real-time PCR and Western blot. Purified CD4^+^T lymphocytes were stimulated by anti-CD3 and anti-CD28 Abs and harvested at the indicated time points. Samples were collected and subjected to Q-PCR analysis. Data were normalized to β-actin expression. Cell lysates were prepared and MCPIP1 protein level was evaluated by Western blot. (B). Detection of MCPIP2, MCPIP3 and MCPIP4 level by real-time PCR. Purified CD4^+^T lymphocytes were stimulated by anti-CD3 and anti-CD28 Abs and harvested at the indicated time points. Samples were collected and subjected to Q-PCR analysis. Data were normalized to β-actin expression. Data were presented as mean ± S.D of three representative independent experiments.

Previous studies have demonstrated that MCPIP1 plays an important role in regulating cytokines in the activation of macrophages through degrading mRNA via a non-ARE conserved element within the 3′UTR [17]. We hypothesized that MCPIP1 may also participate in regulating T cell-derived cytokines in the adaptive immunity. Firstly, we tested if MCPIP1 and other members of the MCPIP family were involved in the activation of T lymphocytes. Purified CD4^+^T and CD8^+^T lymphocytes from spleen and lymph nodes were stimulated with anti-CD3 and anti-CD28 Abs and cells were subjected to Q-PCR or Western blot assay. Q-PCR analysis revealed that MCPIP1 mRNA quickly increased at 3 h and then declined after 12 h both in CD4^+^ and CD8^+^ T cells upon stimulation (Figure 1A and Figure S1A). Its protein was also increased at 6 h and then declined at 24 h upon stimulation in CD4^+^T cells (Figure 1A). MCPIP2, MCPIP3 and MCPIP4 were also induced to some extent with anti-CD3 and antiCD28 stimulation, but their mRNA levels were much lower than that of MCPIP1 (Figure 1B). Moreover, the response of MCPIP1 mRNA level to PMA and ionomycin was similar to anti-CD3 and CD28 Abs stimulation (Figure S1B). These results suggested that MCPIP1 may be involved in the T lymphocytes activation.

### MCPIP1 Negatively Regulates IL-2 during Mouse T lymphocytes Activation

**Figure 2:**
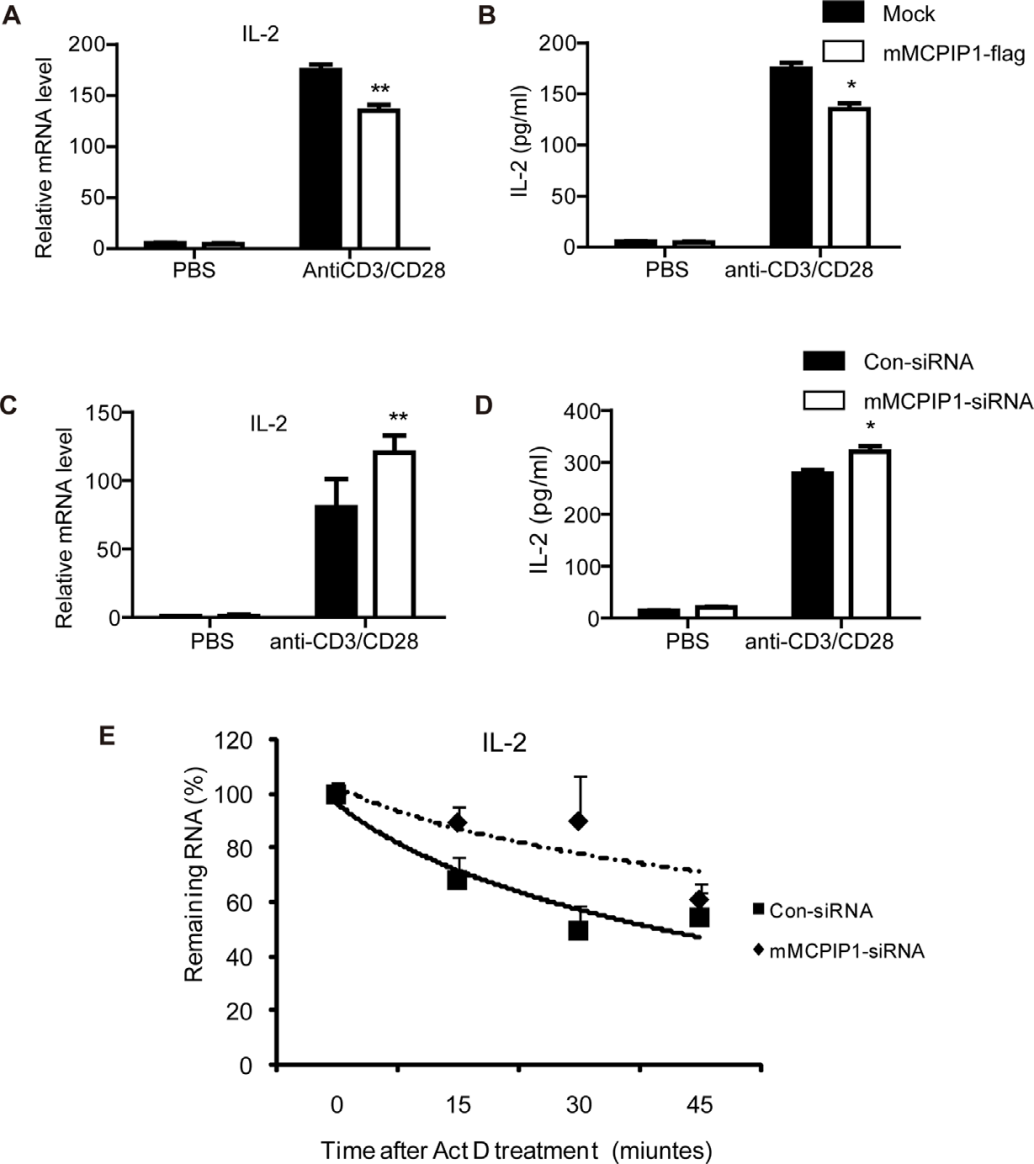
MCPIP1 negatively regulate IL-2 expression in the mouse CD4^+^T lymphocytes. (A–B). Overexpression of MCPIP1 inhibits IL-2 expression. Purified CD4^+^T lymphocytes were transiently transfected with mMCPIP1-flag or control plasmids by electroporation. After incubated for 4 h, cells were challenged with anti-CD3 and anti-CD28 Abs for 12 hours. Cells and cultured medium were harvested and IL-2 mRNA level was measured by Q-PCR (A) and protein level was detected by ELISA (B). (C–D). Knockdown of MCPIP1 promotes IL-2 expression. Purified CD4^+^T lymphocytes were transiently transfected with MCPIP1-siRNA or control-siRNA by electroporation. After incubated for 4 h, cells were challenged with anti-CD3 and anti-CD28 Abs for 12 hours. Cells and cultured media were harvested and IL-2 mRNA level was measured by Q-PCR (C) and protein level was detected by ELISA (D). (E). MCPIP1 destabilizes IL-2 mRNA in mouse CD4^+^T lymphocytes**.** Purified CD4^+^T lymphocytes were transiently transfected with MCPIP1-siRNA or control-siRNA by electroporation. After incubated for 4 h, cells were challenged with anti-CD3 and anti-CD28 Abs. After 6 hours, actinomycin D (10 µg/ml) was added to stop transcription, and total RNA was harvested after 0, 15, 30, or 45 min. IL-2 mRNA levels were measured by Q-PCR and normalized to β-actin mRNA. The normalized level of IL-2 mRNA at time 0 was set at 100. Data were presented as mean ± S.D of three independent experiments. *P<0.05; **P<0.01.

IL-2 is a key cytokine for T lymphocytes survival and proliferation and is quickly induced after T cell activation. As shown in Figure 1, MCPIP1 transcript quickly increased by TCR signaling or PMA stimulation in the primary T lymphocytes, implying that MCPIP1 may regulate the decay of important cytokines, such as IL-2, in the early T- cell immune response. To determine whether IL-2 mRNA was regulated by MCPIP1, mouse primary CD4^+^T lymphocytes were transiently transfected with mMCPIP1-flag or control plasmid by electroporation. After 4 hours of incubation, cells were challenged with anti-CD3 and CD28 Abs for 12 h. Overexpression of MCPIP1 was confirmed by quantitative PCR and Western blot (Figure S2A, B). Q-PCR analysis revealed that IL-2 mRNA was significantly declined with MCPIP1 overexpression (Figure 2A). ELISA assay also showed that IL-2 protein level was decreased, indicating that MCPIP1 overexpression down-regulated IL-2 (Figure 2B).

Next to confirm this notion, we examined IL-2 expression in activated mouse CD4^+^T lymphocytes through siRNA silencing to MCPIP1. The knockdown of MCPIP1 by siRNA was confirmed by Q-PCR and Western blot (Figure S2C, D). As shown in Figure 2C, D, knockdown of MCPIP1 led to an increase of IL-2 mRNA and protein level in the CD4^+^T lymphocytes. Collectively, these data demonstrated that MCPIP1 down-regulated IL-2 during mouse T lymphocytes activation.

To further explore this effect was due to affecting the rate of decay of IL-2 transcript, mouse CD4+T cells, which were transfected with MCPIP1-siRNA, were stimulated by anti-CD3 and CD28 Abs for 6 hours. Then actinomycin D was added to stop transcription. Total RNA was harvested after 0, 15, 30 and 45 minutes and was subjected to Q-PCR. Our result revealed that knockdown of MCPIP1 delayed the decay of IL-2 mRNA (Figure 2E), suggesting that MCPIP1 down-regulated IL-2 gene expression via destabilized its mRNA.

### IL-2 mRNA 3'UTR Responds to MCPIP1 Activity by non-ARE Region

**Figure 3:**
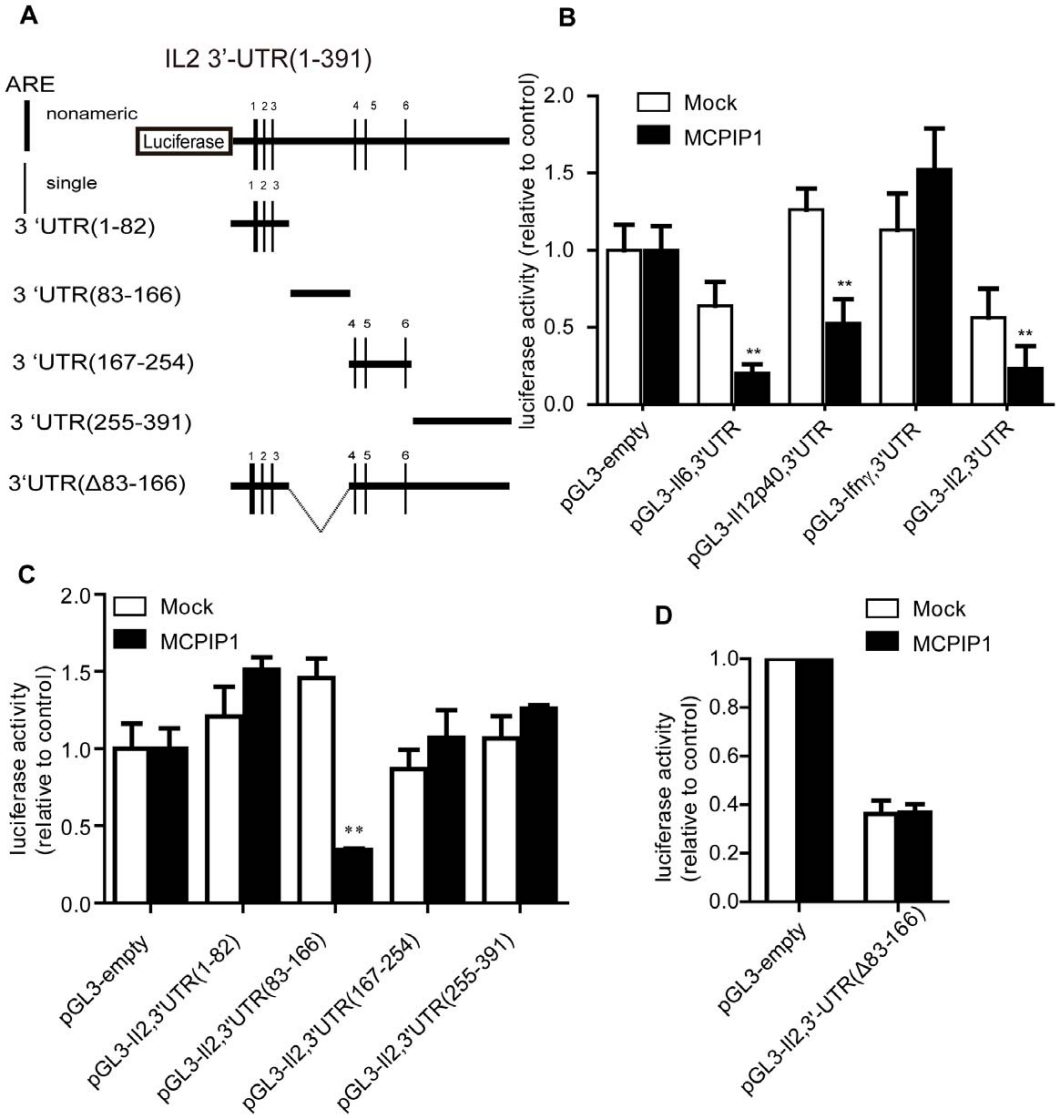
IL-2 mRNA 3'UTR responses to MCPIP1 activity by non-ARE region. (A).Schematic of the IL-2-3′UTR with the indicated AREs and the luciferase report constructs. (B–D). HEK293 cells were co-transfected with pGL3 containing (B) *IL-2*-3′UTR, *IL-6*-3′UTR, *IL-12p40*-3′UTR or *IFN-γ*-3′UTR, (C) indicated sequences of *IL-2*-3′UTR, (D) *IL-2*-3′UTR (Δ83-166).The luciferase activity was determined after 48 h. Data were presented as mean ± S.D of five independent experiments. *P<0.05; **P<0.01.

As shown in Figure 3A, IL-2 3′UTR contains five single AREs and one nonameric ARE. Moreover, there are two conserved regions, one between ARE3 and ARE4, the other after ARE6. MCPIP1 has been recently identified as an RNase, which down-regulates IL-6 mRNA via its binding to a conserved element in the 3′UTR of IL-6 mRNA. Based on the 3′UTR sequence analysis, we speculate that MCPIP1 may regulate IL-2 mRNA decay via an ARE independent pathway. To test this possibility, a series of luciferase reporter plasmids (pGL3) containing a full-length *IL-2* 3′UTR (1-391), *IL-6* 3′UTR (1-403), *IL-12p40* 3′UTR (1-763) and *IFN-γ* (1-631) were co-transfected in HEK293 cells using a pCMV-Tag2b-MCPIP1 plasmid. As reported previously, co-expression of MCPIP1 decreased the luciferase activity of pGL3-*IL-6* 3′UTR and pGL3-*IL-12p40* 3′UTR, but not the pGL3-*IFN-γ* 3′UTR [17]. As expected, we found that the luciferase activity of pGL3*-IL-2* 3′UTR was significantly reduced in the presence of MCPIP1, further confirming that IL-2 may be a target of MCPIP1 (Figure 3B). To identify the possible MCPIP1 target in the IL-2 3′UTR, we constructed a series of luciferase reporter plasmids with several regions of IL-2 3′UTR. The results showed that co-expression of MCPIP1 reduced the luciferase activity of pGL3-*IL-2* 3′UTR (83-166), which is a conserved element between ARE3 and ARE4. Luciferase activities of other regions, including pGL3-*IL-2* 3′UTR (1-82, ARE1 to ARE3), pGL3-*IL-2* 3′UTR (166-254, ARE4 to ARE6) and pGL3-*IL-2* 3′UTR (255-391, the conserved element after ARE6), were not affected compared to the mock control (Figure 3C). Furthermore, the deletion analysis showed that the luciferase activity of pGL3-*IL-2*-3′UTR (Δ83-166) was not altered by MCPIP1 co-expression (Figure 3D). These results indicated that MCPIP1 potentially regulated IL-2 mRNA decay via a conserved element between ARE3 and ARE4 of the 3′UTR.

**Figure 4:**
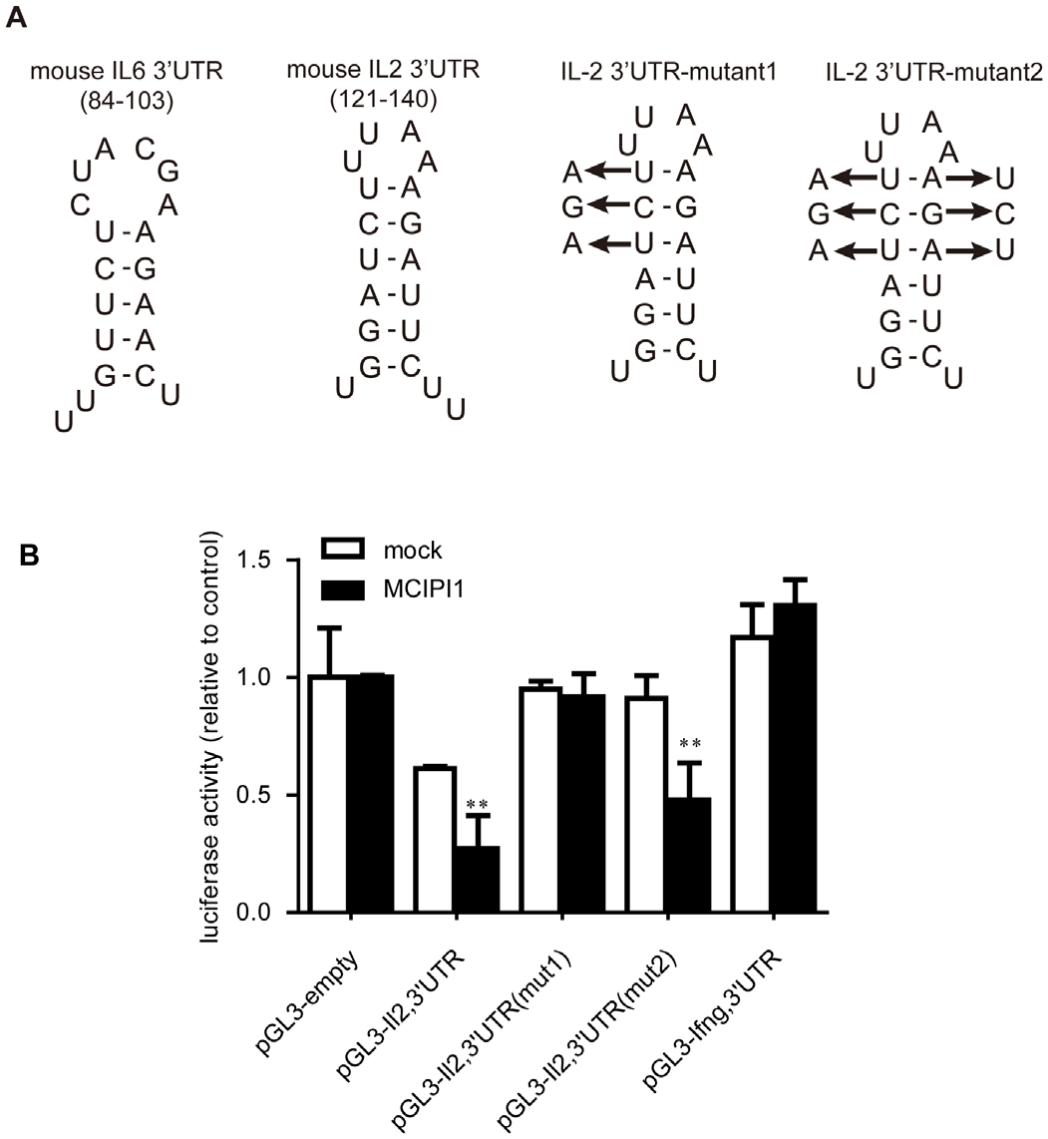
MCPIP1 targets a stem-loop structure in the 83-166 region. (A). Comparison of the *IL-6*-3′UTR’s stem-loop structure with the predicted stem-loop structure of IL-2-3′UTR (left) and the mutation strategy (right). (B). HEK293 cells transfected with pGL3 vector containing sequence as in Fig4A (mutant1 or mutant2) together with the mMCPIP1-flag or control plasmid. The luciferase activity was determined after 48 h.

It has been reported that MCPIP1 degrades IL-6 mRNA through targeting a stem-loop structure [24]. Hence, we analyzed the target element in the IL-2 mRNA 3′UTR (83-166) by M-fold algorithm [25] and found that the 3′UTR formed a stem-loop structure similar to IL-6 mRNA 3′UTR (Figure 4A). We generated two stem-loop mutants to investigate whether this stem-loop structure was the MCPIP1 target. As shown in Figure 4B, disruption of the stem-loop in mutant 1 abolished the changes of the MCPIP1-mediated luciferase activity. However, the luciferase activity in mutant 2, in which the stem-loop structure remains, showed a similar pattern as to the original 3′UTR. These data support that MCPIP1 targets a stem-loop structure in IL-2 3′UTR.

### MCPIP1 Down-regulates IL-2 during Human Peripheral T lymphocytes Activation

Next we compared the sequences of IL-2 mRNA 3′UTR among various species including Mus_musculus, Rattus_norvegius, Bos_taurus, Pan _troglodytes and Homo_sapiens. An evolutionally conserved element (∼80 bp) similar to mouse IL-2 mRNA 3′UTR (83-166) was identified using JalView program [25] (Figure S3) and it also could formed a stem-loop structure with the same stem as mouse IL-2 3′UTR (data not shown). This conserved element may be the target of MCPIP1 in IL-2 regulation.

Because of the importance of IL-2 in the adaptive immune response, we asked whether MCPIP1 had similar effect on regulating IL-2 expression in human peripheral blood CD4^+^T lymphocytes. Firstly, we detected MCPIP1 expression and its response to stimulation of anti-CD3 and CD28 Abs in CD4^+^T lymphocytes isolated from human peripheral blood. Our results demonstrated that MCPIP1 mRNA was detectable (C_T_  = 23) and displayed a mild increase within 3 h of stimulation. The MCPIP1 protein level was also increased, confirmed by Western blot (Figure S4A, B).

**Figure 5:**
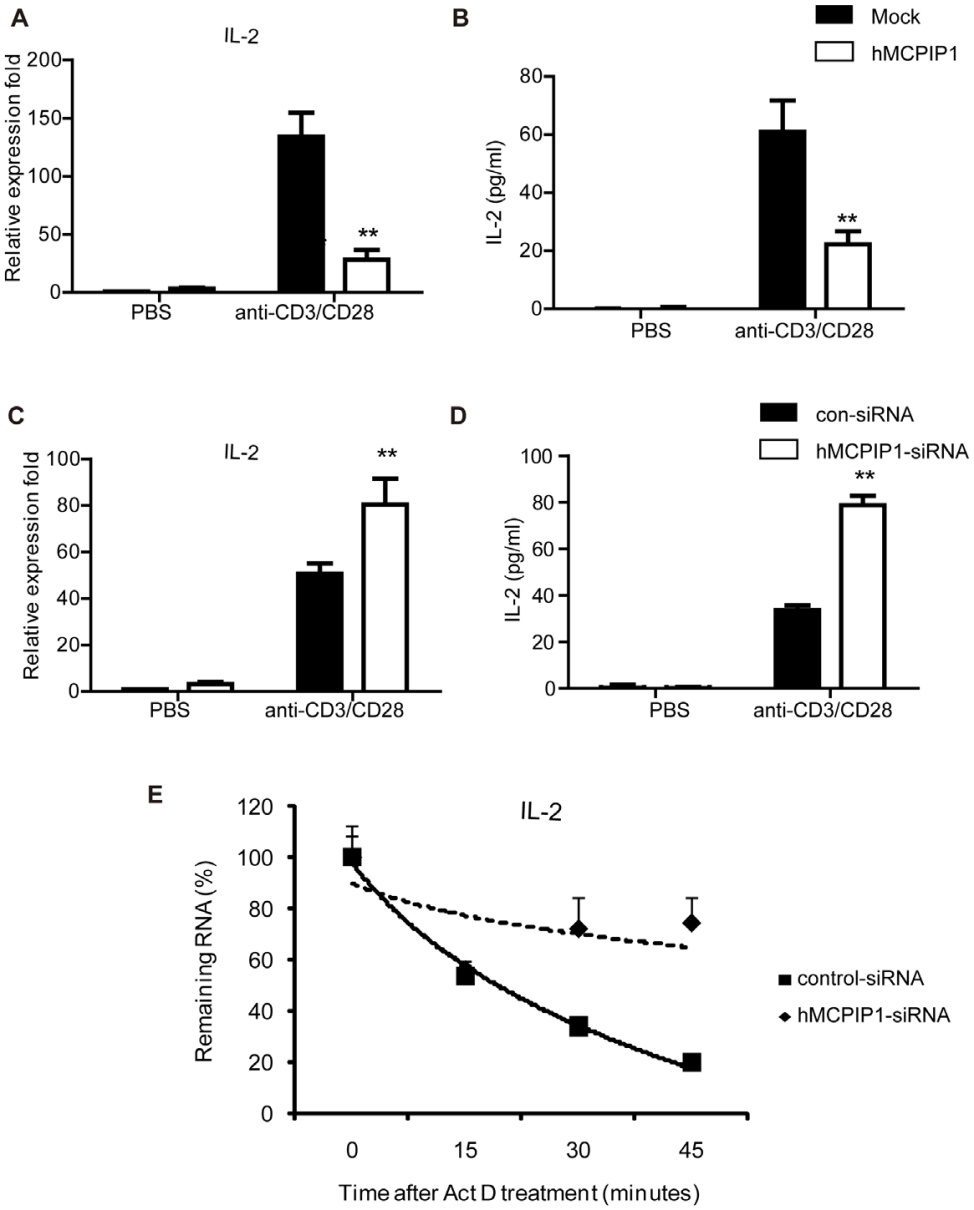
MCPIP1 negatively regulates IL-2 gene expression in human peripheral CD4^+^T lymphocytes. (A–B). Overexpression of MCPIP1 inhibits IL-2 expression. Human peripheral mononuclear cells (PBMC) were obtained from healthy subjects by Ficoll-Hypaque density centrifugation. Purified CD4^+^T lymphocytes were transiently transfected with hMCPIP1-flag or control plasmids by electroporation. After incubated for 4 h, cells were challenged with anti-CD3 and anti-CD28 for 12 hours. Cells and cultured media were harvested and IL-2 mRNA level was measured by Q-PCR (A) and protein level was detected by ELISA (B). (C-D).MCPIP1 Knockdown promotes IL-2 expression stimulated by anti-CD3 and anti-CD28 Abs. Purified CD4^+^T lymphocytes were transiently transfected with control-siRNA or MCPIP1-siRNA by electroporation. After incubated for 4 h, cells were challenged with anti-CD3 and anti-CD28 Abs for 12 hours. Cells and cultured media were harvested and IL-2 mRNA level was measured by Q-PCR (C) and protein level was detected by ELISA (D). (E). MCPIP1 destabilizes IL-2 mRNA in human CD4^+^T lymphocytes**.** Purified CD4^+^T lymphocytes were transiently transfected with MCPIP1-siRNA or control-siRNA by electroporation. After incubated for 4 h, cells were challenged with anti-CD3 and anti-CD28 Abs. 6 h later, actinomycin D (10 µg/ml) was added to stop transcription, and total RNA was harvested after 0, 15, 30, or 45 min. IL-2 mRNA levels were measured by Q-PCR and normalized to β-actin mRNA. The normalized level of IL-2 mRNA at time 0 was set at 100. Data were presented as mean ± S.D of three independent experiments. *P<0.05; **P<0.01.

Then we performed the overexpression and knockdown assays in human CD4^+^T lymphocytes to determine whether MCPIP1 down-regulates IL-2 expression. The overexpression and knockdown were confirmed by Q-PCR and Western blot (Figure S4C, D). The results showed that overexpression of MCPIP1 caused a sharp decline of IL-2 (Figure 5A, B), whereas knockdown of MCPIP1 caused increases of both IL-2 mRNA and protein levels (Figure 5C, D). As shown in Figure 5E, knockdown of MCPIP1 in human CD4+T cells also delayed the decay of IL-2 mRNA. These results were consistent with those of mouse lymphocytes, supporting the hypothesis that MCPIP1 plays a key role in IL-2 mRNA decay.

### MCPIP1 may Bind to IL-2 3′UTR

**Figure 6:**
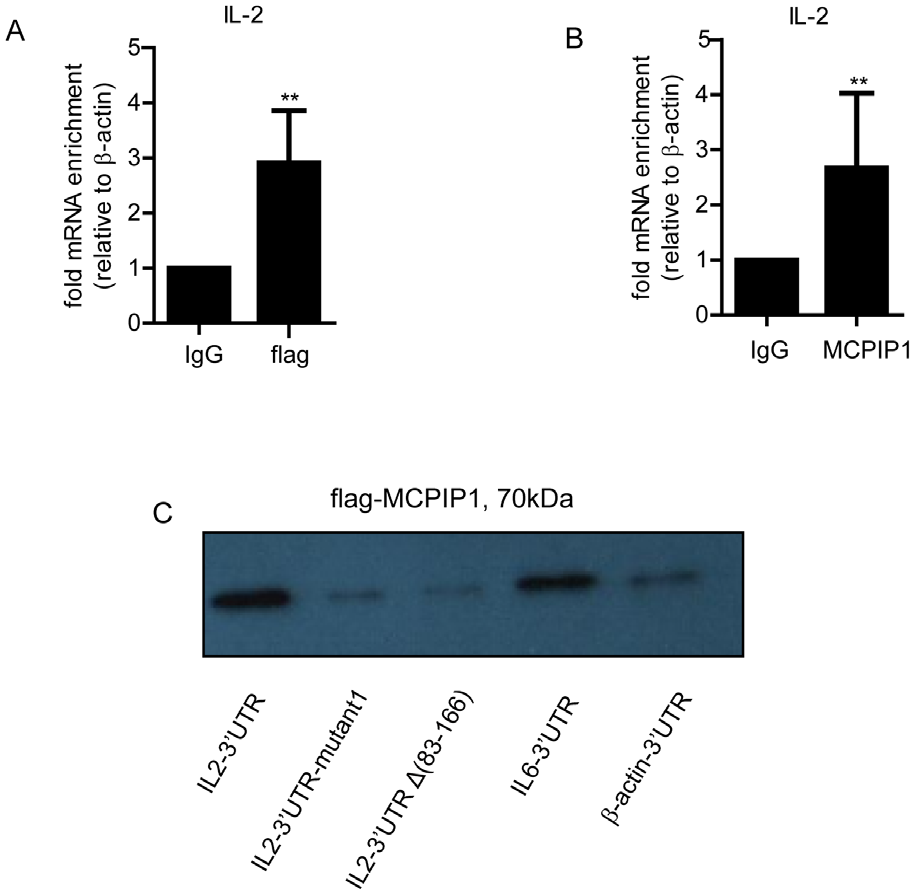
MCPIP1 binds to the 3′UTR of IL-2 mRNA. (A). EL-4 cells were transfected with mMCPIP1- flag or control plasmids. After 24 h, cells were stimulated with PMA (5 ng/ml) and ionomycin (500 ng/ml) for 3 h. Cell lysates were subjected to RIP with anti-flag antibody or IgG. Immumoprecipitated RNA was reverse transcribed to cDNA and then quantified using Sybr Green Q-PCR. (B). Purified mouse T cells were stimulated with anti-CD3 (1 µg/ml) and anti-CD28 (3 µg/ml) antibodies for 6 h. Cell lysates were subjected to RIP with anti-MCPIP1 antibody or IgG. Immumoprecipitated RNA was reverse transcribed to cDNA and then quantified using Sybr Green Q-PCR. (C). MCPIP1-flag expression by Western blot after biotin pulldown assay with biotinylated transcripts - full-length, stem-loop mutant1 and Δ83-166 of IL-2 3′UTR or IL-6 3′UTR as positive control and β-actin 3′UTR as negative control.Data were presented as mean±S.D of three independent experiments. *P < 0.05; **P < 0.01.

To examine whether MCPIP1 could bind to the IL-2 mRNA, we performed RNA-binding protein immunoprecipitation (RIP) experiment on EL-4 cells, which can secret IL-2 after stimulation. EL-4 cells were transiently transfected with mMCPIP1-flag and then stimulated with PMA and Ionomycin for 3 h. Cell lyses were subjected to RIP with anti-flag or IgG antibody to precipitate mRNA. Reverse transcription and Q-PCR analyses showed that anti-flag antibody pulled down larger amount of IL-2 mRNA than IgG did (Figure 6A), implying that MCPIP1 may directly interact with IL-2 mRNA. Then we performed RIP in the mouse T cells which were stimulated with anti-CD3 or CD28 Abs for 6h and found that the anti- MCPIP1 antibody could precipitate more IL-2 mRNA than IgG did as shown in Figure 6B, suggesting MCPIP1 could modestly bind IL-2 mRNA 3′UTR in vivo.

We further validated the interaction of MCPIP1 with the 3′UTR of IL-2 mRNA by biotin pulldown assay. Biotinylated transcripts containing full-length, Δ83-166 and stem-loop mutant1 of IL-2 3′UTR or the full-length of β-actin 3′UTR as negative control and IL-6 3′UTR as positive control were incubated with cell lysates from the HEK293 cells, which were transiently transfected with MCPIP1-flag plasmid. Western blot analysis revealed that MCPIP1 was detected in the sample with the full-length of IL-2 and IL-6 3′UTR biotin probes, whereas the protein lysates precipitated by Δ83-166, stem-loop mutant1 of IL-2 were comparable with the background of β-actin 3′UTR (Figure 6C).

Taken together, these results suggest that MCPIP1 could modestly bind IL-2 3′UTR and this binding was associated with the stem-loop structure.

## Discussion

IL-2, discovered as T cell growth factor, is an essential cytokine for T lymphocytes proliferation and for activating all types of acquired immune responses. Paradoxically, IL-2 appears equally important in limiting immune responses and eliminating autoreactive T lymphocytes [4]. Therefore, IL-2 expression should be precisely regulated at different levels to sustain a steady level for proper immune response.

The mechanism of posttranscriptional regulation is to control the stability of mRNA [26]. The elements of regulating mRNA decay are mainly within the 3′ UTR of IL-2 [7]. About 60 CCCH zinc finger proteins in mouse and human have been identified and some of those have been shown to regulate mRNA expression through binding 3′UTR and affect mRNA stability [15]. TTP is a representative protein that contains two tandem CCCH-zinc fingers. It binds to the AREs of the target transcript’s and is known to regulate the decay of cytokines such as GM-CSF, TNF-α in the macrophages [9], [27], [28] and IL-2, IL-17 in the T lymphocytes [11], [29].

MCPIP1 also belongs to this family and is first identified when induced by MCP-1 in human monocytes. Similarly, it is shown that MCPIP1 controls mRNA stability of inflammatory cytokines, such as IL-6 and IL-12, in the activation of macrophages by targeting a stem-loop structure in non-ARE of 3′UTR as an RNase. Our data demonstrated that MCPIP1 down-regulated IL-2 mRNA via a conserved non-ARE domain in the IL-2 3′UTR of T lymphocytes. This was different from the mechanism of TTP, recruits deadenylases to remove polyA tails and its function of degrading mRNA is mediated by the exosome in the 3′-5′ direction [27]. However, MCPIP1 binds to a conserved element in 3′UTR and it has a PIN-like domain with endo/exonuclease activity [17]. We also showed that MCPIP1 directly bound to IL-2 mRNA and targeted a stem-loop structure in its 3′UTR. All these data indicated that MCPIP1 was involved in the IL-2 posttranscriptional regulation in an ARE-independent pathway. Previous studies have shown that MCPIP1 targets the terminal loop of pre-miRNAs or IL-6 3′UTR and different lengths of stem or loop does not affect its function [19], [24]. It is may explain that although there’s 2 nt difference in the mouse and human IL-2 loop structure, MCPIP1 could still regulate IL-2 in human CD4+T cells. We could not, however, exclude the possibility that there exist other targets of MCPIP1 in T cells besides IL-2. We found that the RNA binding protein NF90, which can bind to the 3′UTR and slow down the degradation of IL-2 mRNA [13], was also down-regulated by MCPIP1 while MCPIP1 knockdown had opposite effect (Figure S5A). Further results showed that MCPIP1 decreased the luciferase activity when co-transfected with the full-length 3′UTR of NF90 (Figure S5B) and it directly bound to the mRNA of NF90 (Figure S5C). These results suggest that MCPIP1 may regulate IL-2 indirectly. Besides, Hiroshi I. et al. have identified that MCPIP1 can suppress miRNA activity and biogenesis through antagonizing Dicer to degrade pre-microRNA [19].

Another possibility is that MCPIP1 participates in the regulation of IL-2 gene at transcription level. MCPIP1 has a putative nuclear localization signal sequence and a single zinc finger domain for DNA or RNA-binding, which suggest that it may act as a transcription factor [14]. It has been reported that MCPIP1 inhibited the promoter activity of TNF-α and iNOS [14], [16]. Further studies indicate that MCPIP1 negatively regulates JNK and NF-κB signaling as a deubiquitinase targeting TRAF family in the activation of macrophages [18]. It is also possible MCPIP1 may regulate IL-2 via its negative regulation of NF-κB, which is involved in the IL-2 gene regulation.

Here, we report that MCPIP1 modestly binds to a conserved element of IL-2 3′UTR, leading to its mRNA decay via an ARE-independent mechanism. Our study also suggests that MCPIP1 could be potentially used as drug target for many of the inflammatory diseases. Further studies will be focused on the exact targets of MCPIP1, and the regulation mechanism of MCPIP1 in T lymphocytes.

## Supporting Information

Figure S1 **MCPIP1 are induced in mouse primary CD4^+^and CD8^+^T lymphocytes.** (A).Purified CD8^+^T lymphocytes were stimulated by anti-CD3 and anti-CD28 Abs and harvested at the indicated time points. Samples were collected and subjected to quantitative PCR analysis. (B).Purified CD4^+^T or CD8^+^T lymphocytes were stimulated by PMA and Ionomycin and harvested at the indicated time points. Samples were collected and subjected to quantitative PCR analysis. Data are the mean±S.D. of three independent experiments.*P<0.05; **P<0.01.
(TIF)

Figure S2 **MCPIP1 negatively regulate IL-2 expression in the mouse CD4^+^T lymphocytes.** (A-B).Purified CD4^+^T lymphocytes were transiently transfected with mMCPIP1-flag or control plasmids by electroporation. After resting for 4 h, cells were challenged with anti-CD3 and anti-CD28 Abs for 12 hours. The MCPIP1 overexpression was detected by Q-PCR and Western blot using anti-flag antibody. (C-D).Purified CD4^+^T lymphocytes were transiently transfected with MCPIP1-siRNA or control-siRNA by electroporation. After resting for 4 h, cells were challenged with anti-CD3 and anti-CD28 Abs for 12 hours. The MCPIP1 knockdown efficiency was detected by Q-PCR and Western blot.
(TIF)

Figure S3 **Sequence comparison of the IL-2-3′UTR.** The full length of the 3′UTR from various species were aligned by ClustalW and the conservation (below the alignment) was analyzed by the online JalView program (http://www.ebi.ac.uk/clustalw/index.html). The 83-166 region of mouse IL-2-3′UTR and the corresponding sequences were highlighted.
(TIF)

Figure S4 **MCPIP1 are induced in the human primary CD4^+^ T lymphocytes and negatively regulates IL-2 gene expression in human peripheral CD4^+^T lymphocytes.** (A-B).Isolation CD4^+^T lymphocytes from human peripheral blood monocytes were stimulated by anti-CD3 and anti-CD28 Abs and harvested at the indicated time points. Samples were collected and subjected to quantitative PCR analysis. Data are from three independent experiments and normalized to β-actin expression. Protein level of MCPIP1 was determined by Western blot. (C–D).Human peripheral mononuclear cells (PBMC) were obtained from healthy subjects by Ficoll-Hypaque density centrifugation. Purified CD4+T lymphocytes were transiently transfected with hMCPIP1-flag or MCPIP1-siRNA by electroporation. MCPIP1 overexpression was detected by Western blotting using anti-flag antibody. The MCPIP1 knockdown efficiency was detected by Q-PCR.
(TIF)

Figure S5 **MCPIP1 negatively regulates NF90 in CD4^+^T lymphocytes through directly binding 3′UTR.** (A).Purified CD4^+^T lymphocytes were transiently transfected with mMCPIP1-flag or control plasmids, and MCPIP1-siRNA or control-siRNA by electroporation. After resting for 4 h, cells were challenged with anti-CD3 and anti-CD28 Abs for 12 hours. Cells were harvested and NF90 mRNA level was measured by Q-PCR. (B). HEK293 cells were co-transfected with pGL3 containing NF90-3′UTR and the mMCPIP1-flag or control plasmids. The luciferase activity was determined after 48 h. (C). EL-4 cells were transfected with mMCPIP1- flag or control plasmids. After 24 h, cells were stimulated with PMA (5 ng/ml) and ionomycin (500 ng/ml) for 3 h.Then cell lysates were subjected to RIP with anti-flag antibody or IgG. Immumoprecipitated RNA was reverse transcripted to cDNA and then quantified using Sybr Green qPCR. Data are expressed as fold enrichment relative to IgG. Data are the mean±S.D (n = 3) of three independent experiments. *P<0.05; **P<0.01.
(TIF)
