# Supplementary figures and images for: MCPIP1 Down-Regulates IL-2 Expression through an ARE-Independent Pathway
Source: PLoS One. 2012 Nov 21;7(11):e49841. doi: 10.1371/journal.pone.0049841 (PMC3504106; doi:10.1371/journal.pone.0049841)

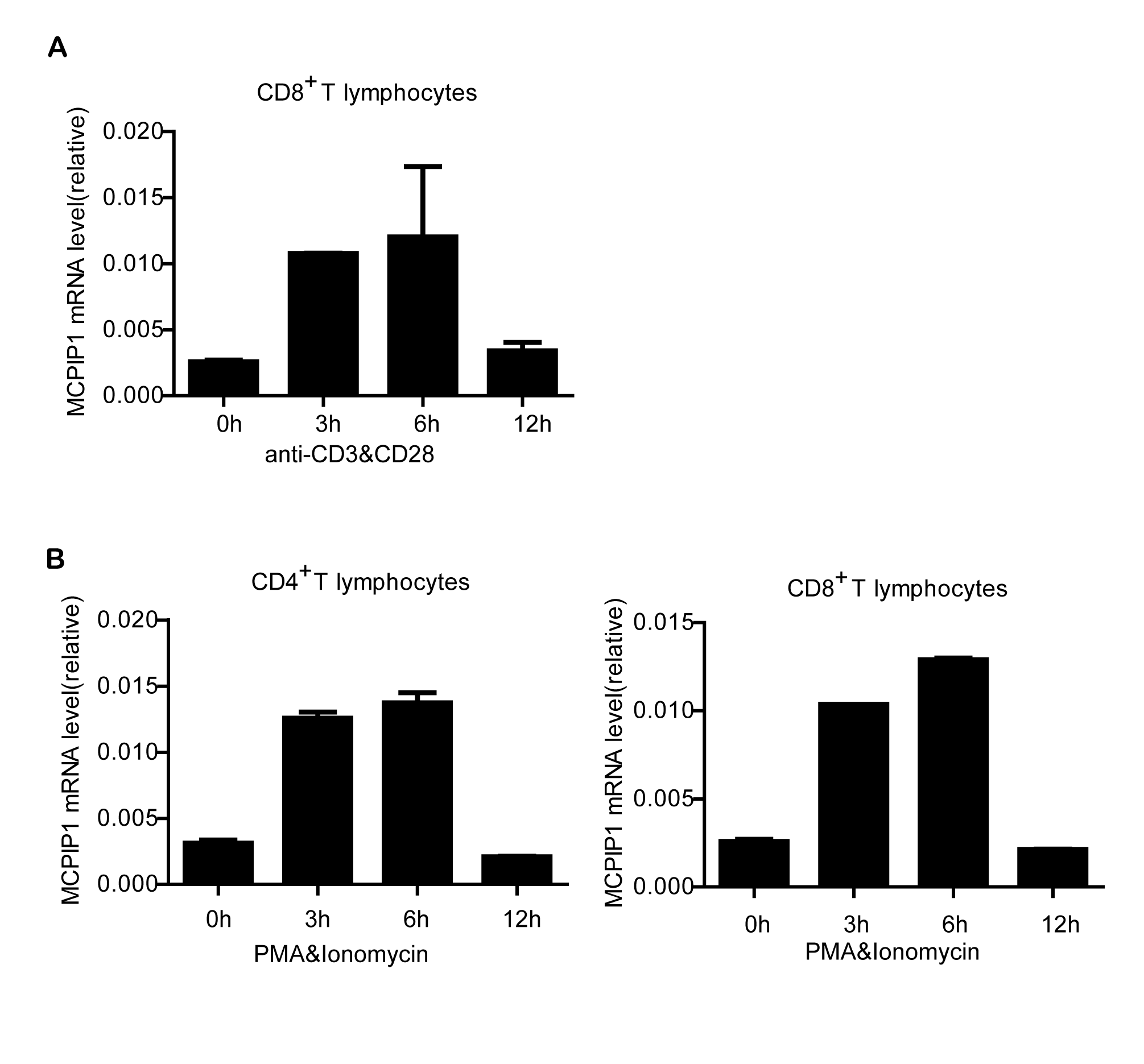

Supplement: Figure S1 — MCPIP1 are induced in mouse primary CD4+and CD8+T lymphocytes. (A).Purified CD8+T lymphocytes were stimulated by anti-CD3 and anti-CD28 Abs and harvested at the indicated time points. Samples were collected and subjected to quantitative PCR analysis. (B).Purified CD4+T or CD8+T lymphocytes were stimulated by PMA and Ionomycin and harvested at the indicated time points. Samples were collected and subjected to quantitative PCR analysis. Data are the mean±S.D. of three independent experiments.*P<0.05; **P<0.01. (TIF) [file pone.0049841.s001.tif]

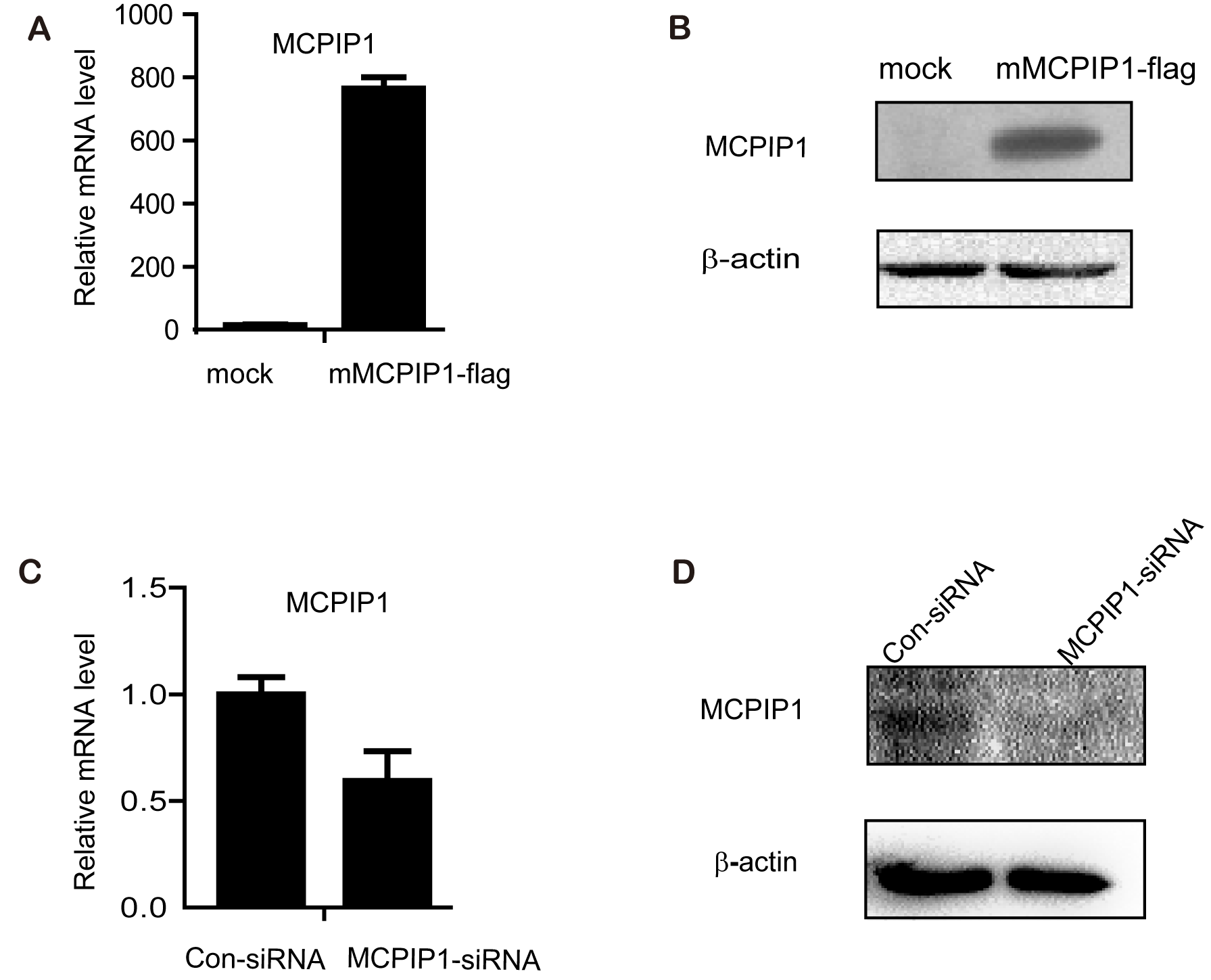

Supplement: Figure S2 — MCPIP1 negatively regulate IL-2 expression in the mouse CD4+T lymphocytes. (A-B).Purified CD4+T lymphocytes were transiently transfected with mMCPIP1-flag or control plasmids by electroporation. After resting for 4 h, cells were challenged with anti-CD3 and anti-CD28 Abs for 12 hours. The MCPIP1 overexpression was detected by Q-PCR and Western blot using anti-flag antibody. (C-D).Purified CD4+T lymphocytes were transiently transfected with MCPIP1-siRNA or control-siRNA by electroporation. After resting for 4 h, cells were challenged with anti-CD3 and anti-CD28 Abs for 12 hours. The MCPIP1 knockdown efficiency was detected by Q-PCR and Western blot. (TIF) [file pone.0049841.s002.tif]

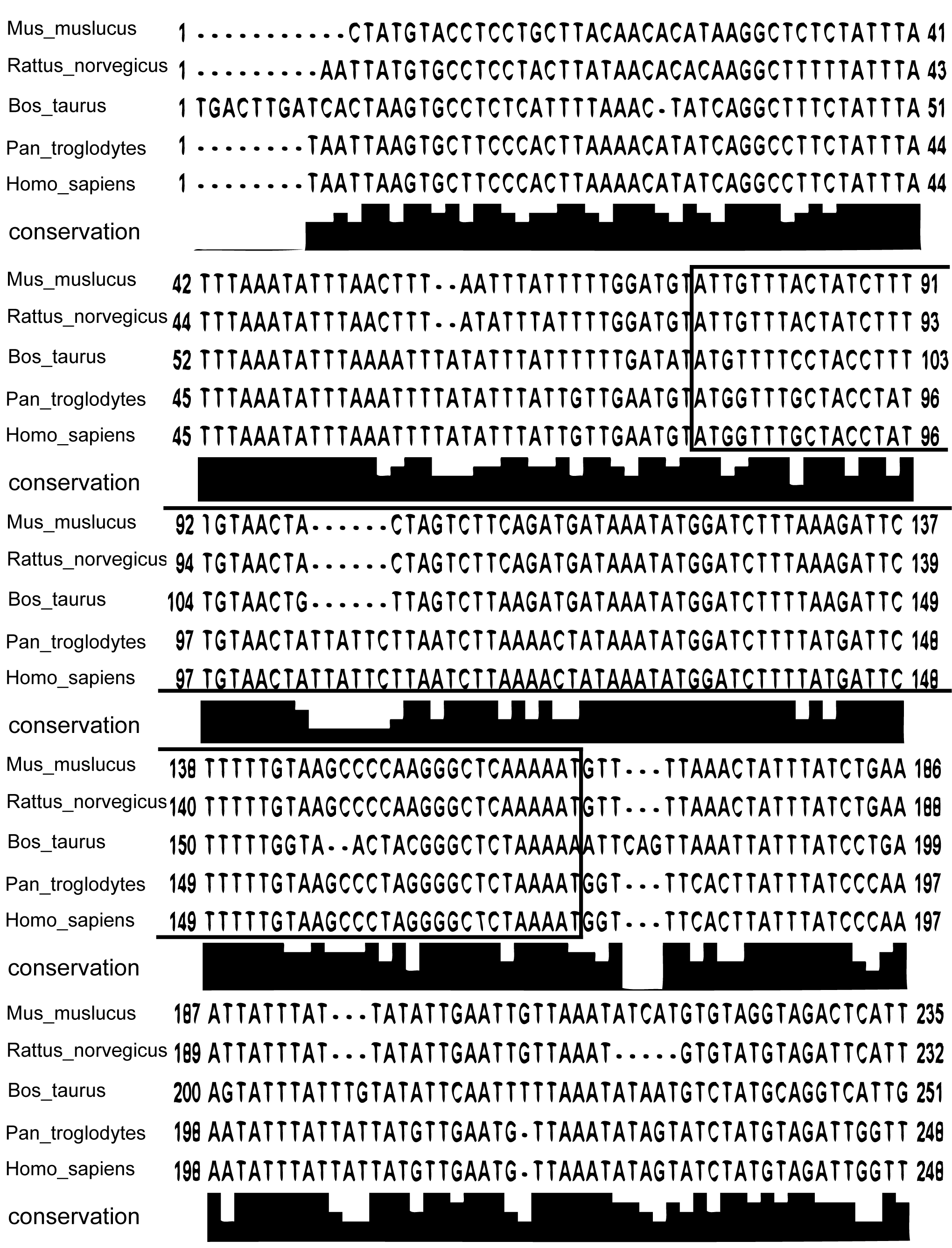

Supplement: Figure S3 — Sequence comparison of the IL-2-3′UTR. The full length of the 3′UTR from various species were aligned by ClustalW and the conservation (below the alignment) was analyzed by the online JalView program (http://www.ebi.ac.uk/clustalw/index.html). The 83-166 region of mouse IL-2-3′UTR and the corresponding sequences were highlighted. (TIF) [file pone.0049841.s003.tif]

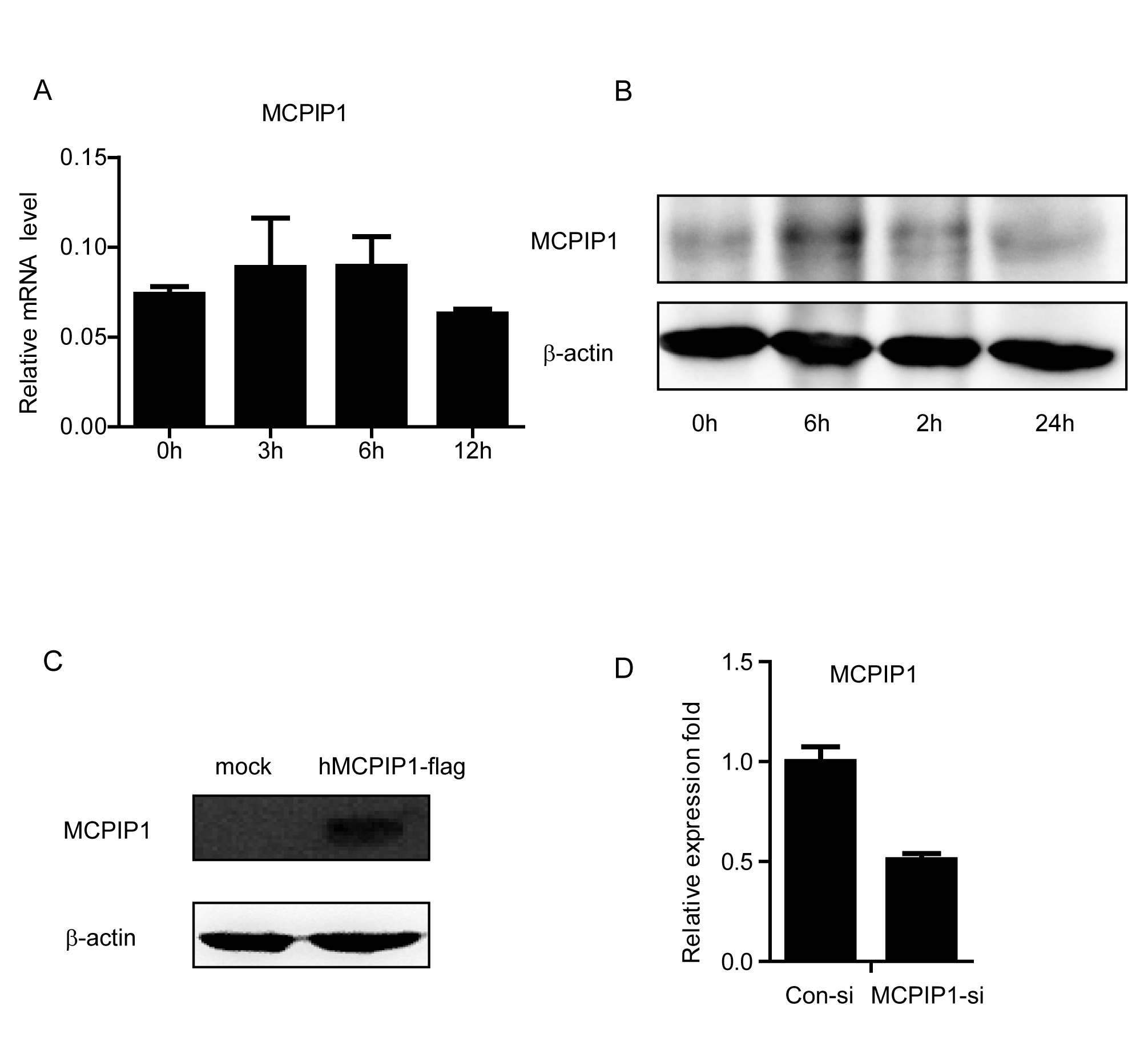

Supplement: Figure S4 — MCPIP1 are induced in the human primary CD4+ T lymphocytes and negatively regulates IL-2 gene expression in human peripheral CD4+T lymphocytes. (A-B).Isolation CD4+T lymphocytes from human peripheral blood monocytes were stimulated by anti-CD3 and anti-CD28 Abs and harvested at the indicated time points. Samples were collected and subjected to quantitative PCR analysis. Data are from three independent experiments and normalized to β-actin expression. Protein level of MCPIP1 was determined by Western blot. (C–D).Human peripheral mononuclear cells (PBMC) were obtained from healthy subjects by Ficoll-Hypaque density centrifugation. Purified CD4+T lymphocytes were transiently transfected with hMCPIP1-flag or MCPIP1-siRNA by electroporation. MCPIP1 overexpression was detected by Western blotting using anti-flag antibody. The MCPIP1 knockdown efficiency was detected by Q-PCR. (TIF) [file pone.0049841.s004.tif]

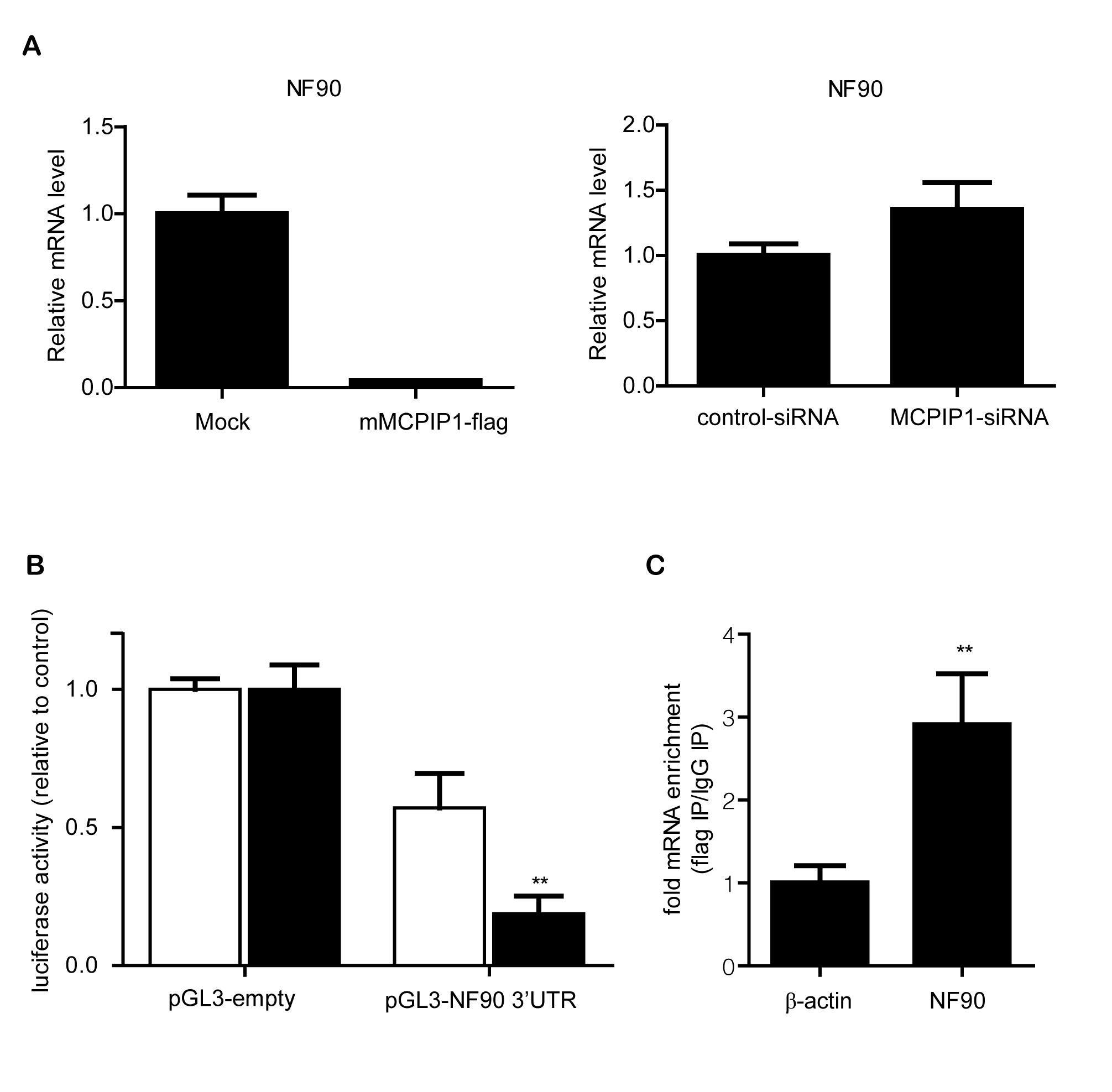

Supplement: Figure S5 — MCPIP1 negatively regulates NF90 in CD4+T lymphocytes through directly binding 3′UTR. (A).Purified CD4+T lymphocytes were transiently transfected with mMCPIP1-flag or control plasmids, and MCPIP1-siRNA or control-siRNA by electroporation. After resting for 4 h, cells were challenged with anti-CD3 and anti-CD28 Abs for 12 hours. Cells were harvested and NF90 mRNA level was measured by Q-PCR. (B). HEK293 cells were co-transfected with pGL3 containing NF90-3′UTR and the mMCPIP1-flag or control plasmids. The luciferase activity was determined after 48 h. (C). EL-4 cells were transfected with mMCPIP1- flag or control plasmids. After 24 h, cells were stimulated with PMA (5 ng/ml) and ionomycin (500 ng/ml) for 3 h.Then cell lysates were subjected to RIP with anti-flag antibody or IgG. Immumoprecipitated RNA was reverse transcripted to cDNA and then quantified using Sybr Green qPCR. Data are expressed as fold enrichment relative to IgG. Data are the mean±S.D (n = 3) of three independent experiments. *P<0.05; **P<0.01. (TIF) [file pone.0049841.s005.tif]
